# Hybrid Deep Learning Approach for Accurate Tumor Detection in Medical Imaging Data

**DOI:** 10.3390/diagnostics13061025

**Published:** 2023-03-08

**Authors:** Mehmet Akif Cifci, Sadiq Hussain, Peren Jerfi Canatalay

**Affiliations:** 1 The Institute of Computer Technology, Tu Wien University, 1040 Vienna, Austria; 2 Department of Computer Engineering, Bandirma Onyedi Eylul University, Balikesir 10200, Turkey; 3 Engineering and Informatics Department, Klaipėdos Valstybinė Kolegija/Higher Education Institution, 92294 Klaipeda, Lithuania; 4 Examination Branch, Dibrugarh University, Dibrugarh 786004, Assam, India; 5 Department of Computer Engineering, Haliç University, Istanbul 34394, Turkey

**Keywords:** electronic medical records, medical event extraction, transfer learning, joint extraction

## Abstract

The automated extraction of critical information from electronic medical records, such as oncological medical events, has become increasingly important with the widespread use of electronic health records. However, extracting tumor-related medical events can be challenging due to their unique characteristics. To address this difficulty, we propose a novel approach that utilizes Generative Adversarial Networks (GANs) for data augmentation and pseudo-data generation algorithms to improve the model’s transfer learning skills for various tumor-related medical events. Our approach involves a two-stage pre-processing and model training process, where the data is cleansed, normalized, and augmented using pseudo-data. We evaluate our approach using the i2b2/UTHealth 2010 dataset and observe promising results in extracting primary tumor site size, tumor size, and metastatic site information. The proposed method has significant implications for healthcare and medical research as it can extract vital information from electronic medical records for oncological medical events.

## 1. Introduction 

This research paper focuses on medical events related to tumors and presents a novel approach to medical event extraction. Our method combines Generative Adversarial Networks (GANs) for data augmentation with the incorporation of pseudo-data production within the algorithm [1].

The primary objective of this study is to improve the precision and generalizability of the machine learning model used for medical event extraction. To achieve this, we propose a two-stage technique consisting of pre-processing and model training. In the pre-processing stage, we cleanse the data by removing extraneous or duplicate information and normalize the text data to standardize its format. We also generate pseudo-data to augment the training dataset. This stage aims to ensure that the data used for model training is of high quality and consistent, enhancing the model’s ability to extract medical events accurately. By implementing our proposed technique, we expect to achieve improved accuracy and generalizability in medical event extraction, which has significant implications for healthcare and medical research. The process employs data augmentation methods and GANs to generate new annotated medical records comparable to the original annotated data.

The collected characteristics from the pre-processing phase are supplied into a Convolutional Neural Network (CNN) with a multi-head attention mechanism [2] during the model training stage. The model is trained using annotated medical records, including accurate annotated data and pseudo-data. We next compare the performance of the trained model to that of the Convolutional Clinical Named Entity Recognition (CCMNN) model utilizing precision, recall, and F1 score. The CCMNN type of CNN was developed primarily for identifying clinically named entities in unstructured medical texts, such as illnesses, symptoms, therapies, and anatomical entities [3]. It extracts pertinent information from the input text and identifies named things using convolutional and max-pooling layers. CCMNN’s architecture is meant to account for the unique peculiarities of medical texts and enhance the Named Entity Recognition (NER) performance in this area [4]. Compared to the CCMNN model, our findings demonstrate a considerable increase in tumor-related medical event extraction performance. In order to improve transfer learning skills for various types of tumor-related medical events, our proposed method also increases the number and types of annotated medical records. In medical event extraction, the combination of CNN, multi-head attention mechanism, GANs for data augmentation, and pseudo-data production constitutes a novel approach. A lack of annotated medical records is one of the most significant obstacles in medical NLP. It is challenging for researchers to collect sufficient annotated data for machine learning models due to the expense and time necessary for annotating big datasets of medical records. Our proposed pseudo-data-generation technique overcomes this challenge by producing pseudo-data from existing annotated data, expanding the number and types of annotated medical data accessible for training and testing. 

In recent years, applying advanced numerical and optimization techniques has significantly contributed to developing machine learning algorithms in various fields. For instance, a numerical method for solving the nonlinear equations of the Emden–Fowler model has been proposed in the context of ocean engineering and science [2]. Another study has utilized optimal control to study the dynamical behaviors of fractional financial awareness models. In medical event extraction, the combination of CNN, multi-head attention mechanism, GANs, and pseudo-data production constitute a novel approach. By producing pseudo-data from existing annotated data, our proposed method overcomes the challenge of limited annotated medical records, expanding the number and types of annotated medical records accessible for training and testing. Furthermore, the proposed method is extensible to many medical domains, enabling the extension of annotated medical records in various medical domains. Specifically, the proposed method aims to (1) offer a standard extraction method for medical events that simultaneously extracts the primary tumor site, size, and metastatic site, intending to overcome the constraints of prior extraction methods, such as CCMNN; (2) enhance the performance of medical event extraction linked to tumors, notably in the extraction of primary tumor size, by comparing our proposed method with CCMNN; and (3) address the issue of limited annotated medical record texts for oncology medical events by proposing a pseudo-data-generation algorithm to increase the number and types of annotated medical records and to enhance transfer learning capabilities for various types of tumor-related medical events.

In this study, a novel approach is proposed for the automatic extraction of the size of primary tumors from medical records. The primary objective of this research is to develop a machine-learning-based approach for the automated extraction of primary tumor size information from medical records. The proposed approach will address the limitations of manual extraction, which can be error-prone and time-consuming. By leveraging machine learning techniques, we aim to develop a more efficient and accurate method for tumor size extraction.

The proposed approach has the potential to significantly impact the field of medical informatics by improving the precision and efficiency of medical event extraction. The successful development of this approach could lead to faster and more accurate diagnoses, improved treatment planning, and ultimately better patient outcomes. In addition, our approach could help healthcare providers to manage patient data better, leading to more effective care delivery.

The significance of this research lies in the potential to make a tangible impact on patient care and outcomes while also addressing an ongoing challenge in the field of medical informatics. We hope to contribute to the ongoing efforts to improve cancer management and patient outcomes by developing a more efficient and accurate method for tumor size extraction.

The benefits of the paper are its originality, relevance, and possible influence on its relevant subject. When the research presents a novel deep learning model or a unique application of an existing model that surpasses earlier techniques in terms of accuracy, efficiency, or scalability, it will have a considerable advantage over other studies.

This research paper presents a novel approach to medical event extraction that focuses on tumors, using Generative Adversarial Networks (GANs) for data augmentation and pseudo-data production within the algorithm to improve the precision and generalizability of the machine learning model. The proposed technique includes pre-processing and model training stages and utilizes a Convolutional Neural Network (CNN) with a multi-head attention mechanism. The proposed approach aims to extract the primary tumor site, size, and metastatic site accurately and efficiently while addressing the issue of limited annotated medical records. The proposed method has the potential to impact patient care and outcomes by improving cancer management and treatment planning.

The paper’s contributions can be summarized as follows:

- This research paper proposes a novel approach to medical event extraction that focuses on tumors using Generative Adversarial Networks (GANs) for data augmentation and pseudo-data production within the algorithm.
- The proposed technique includes pre-processing and model training stages and utilizes a Convolutional Neural Network (CNN) with a multi-head attention mechanism to improve accuracy and generalizability in medical event extraction.
- By cleansing data, normalizing text data, and generating pseudo-data to augment the training dataset, the pre-processing stage aims to ensure the data used for model training is of high quality and consistent.
- The trained model is compared to the Convolutional Clinical Named Entity Recognition (CCMNN) model, demonstrating a considerable increase in tumor-related medical event extraction performance.
- The proposed method overcomes the challenge of limited annotated medical records by producing pseudo-data from existing annotated data, expanding the number and types of annotated medical records accessible for training and testing.
- The proposed approach has the potential to significantly impact the field of medical informatics by improving the precision and efficiency of medical event extraction, leading to faster and more accurate diagnoses, improved treatment planning, and better patient outcomes.
- The significance of this research lies in the potential to make a tangible impact on patient care and outcomes while also addressing an ongoing challenge in the field of medical informatics.

## 2. Related Works

This section presents an overview of related work in the field of medical information extraction (Table 1). We highlight previous studies that have used shallow machine learning techniques, such as Hidden Markov Models (HMMs), Conditional Random Fields (CRFs), and Support Vector Machines (SVMs). In medical information extraction, similar to information extraction in broader domains [5], the process involves identifying professional language boundaries in medical texts and categorizing them based on domain-specific data [6]. Shallow machine learning techniques, including Hidden Markov Models (HMM), are statistical models used to describe sequences of observations. HMMs assume that the observations are generated by a hidden process that is not directly observable but can only be inferred from the observed data. The hidden process is assumed to be a Markov process, meaning that the probability of observing a particular state at any given time depends only on the state observed at the previous time. In an HMM, the hidden process is modeled by a sequence of states, while the observations are modeled by a sequence of output symbols associated with each state. Conditional Random Fields (CRF) and Support Vector Machines (SVM) [7] are often used in medical information extraction as well. In a study using CRF on the JNLPBA 2004 dataset, [8] reported an F1 value of 72.23, while [9] proposed a multi-feature fusion CRF method that detects sickness and symptom entities in medical language as unregistered entity names. In another study, a CRF model was used to recognize biological objects, resulting in an F1 value of 71.39 on the JNLPBA 2004 dataset [10]. The paper [11] was the first to use neural networks to construct word vectors from unlabeled biomedical texts and to design a multi-layer neural network resulting in an F1 value of 71 on the JNLPBA 2004 dataset for the extraction of medical information. On the BioCreativeGM dataset, the BiLSTM model achieved an F1 value of 72.76, whereas [12] achieved an F1 value of 88.6. The authors of [13] proposed a CNN BLSTM CRF-based neural network model and achieved the most excellent F1 value. 

Ref. [14] proposes a standard extraction method for tumor-related medical events that combines the extraction of the primary tumor site and size and tumor metastasis sites. The method uses a key-based approach and a pseudo-data-generation algorithm to improve transfer learning and overcome the small number and types of annotation texts for tumor-related medical events. Ref. [15] presents public general pre-trained word embeddings as input features and employs a hybrid dilation convolution structure and the attention mechanism to improve the two networks. The model achieved a reasonable F1 score of 73.72% on the JNLPBA corpus and is the first to adopt the hybrid structure that combines CNN with BLSTM in BNER. Ref. [16] studies the use of a novel neural network architecture for biomedical named entity recognition (BNER), which involves extracting chemical names from biomedical texts to support research. The proposed system uses bidirectional long short-term memory (BLSTM), dynamic recurrent neural network (RNN), and conditional random field (CRF) with character and word level embedding as the only features. Ref. [17] discusses the importance of NLP technology in the medical field, particularly in event extraction from electronic medical records. The focus of the article is on tumor-related medical event extraction, the method is tested on the CCKS2020 dataset and achieves an F1 value of 73.52.

In [18] the authors study a review of recent research on biometric finger vein recognition systems with a focus on deep learning-based techniques, presentation attack detection methods, and multimodal-based systems. They suggested solutions related to deep learning, PAD, and multimodal-based finger vein recognition systems. Ref. [19] proposed a pattern-based extraction method for tumor-related medical events, which had an F1 value of 69.7 on the i2b2/UTHealth 2010 dataset. Ref. [20] proposed neural network collaboration-based multiple extraction techniques with an F1 value of 76.35 for the i2b2/UTHealth 2010 dataset. On the i2b2/UTHealth 2010 dataset, [21] developed a model-based multi-sequence labeling method with an F1 value of 76.17. Ref. [22] proposed an Elmo-based sequence labeling method that incorporates rules, resulting in an F1 value of 70.69, using the i2b2/UTHealth 2010 dataset. Recently, Ref. [23] proposed an extraction strategy based on Robert for the i2b2/UTHealth 2010 dataset, resulting in an F1 value of 76.23. The BiLSTM-CRF model was applied to the i2b2/UTHealth 2010 dataset, and an F1 value of 76.51 was achieved. Cross-Correlation Multi-Neural Networks (CCMNN) [6] and Generative Adversarial Networks (GAN) [24,25,26] have been widely applied to various image-processing tasks, such as image classification and generation. CCMNN improves the performance of picture classification tasks by utilizing cross-correlation across several neural networks. It has been applied to several applications with promising results. GANs are an efficient technique for generative modeling and have been applied to various computer vision issues, including image synthesis, style transfer, and denoising.

**Table 1:** A Comprehensive Comparison of Medical Information Extraction Approaches.

Technique	Reference	Dataset	F1 Value
Hidden Markov Model (HMM)	[7]	i2b2/UTHealth 2010	79.51
Conditional Random Field (CRF)	[8]	JNLPBA 2004	77.65
Support Vector Machine (SVM)	[9]	i2b2/UTHealth 2010	75.15
Multi-Feature Fusion CRF	[10]	Medical Text	80.96
CRF with Artificial Features	[11]	JNLPBA 2004	81.41
Neural Networks for Word Vectors	[12]	JNLPBA 2004	75.56
BiLSTM	[13]	BioCreativeGM	79.89
JNLPBA 2004	[14]	JNLPBA 2004	79.32
CNN BLSTM CRF Neural Network	[15]	BioCreativeIIGM	76.23
Pattern-Matching-Based Extraction	[16]	i2b2/UTHealth 2010	80.72
Neural-Network Collaboration-Based Extraction	[17]	i2b2/UTHealth 2010	81.19
Multi-Sequence Labeling Model-Based Extraction	[18]	i2b2/UTHealth 2010	79.51
Elmo-Based Sequence Labeling	[19]	i2b2/UTHealth 2010	77.65
RoBERT-Based Extraction	[20]	i2b2/UTHealth 2010	75.15
BiLSTM-CRF	[21]	i2b2/UTHealth 2010	80.96

## 3. Materials and Methods

### 3.1. Task Assessment and Analysis

The primary tumor site, tumor size, and metastasis site are critical features in a cancer patient’s diagnosis, treatment, and prognosis. In medical information extraction, accurate identification, and extraction of these features from clinical narratives are essential for cancer research and clinical decision-making. The primary tumor site is the tissue or organ where a specific malignant tumor originates. It is often referred to using various terms, such as “cancer”, “malignant tumor”, “MT”, and “CA”. Accurately identifying and extracting the primary tumor site is crucial for determining the type of cancer and its severity. The primary tumor size is a measurable evaluation of the dimensions of the primary tumor, often expressed as length, area, or volume. It is an essential clinical indicator that can help determine the cancer stage and the most appropriate treatment strategy. Accurate extraction of the primary tumor size from clinical narratives can significantly aid cancer research and treatment. 

The tumor metastasis site is where a malignant tumor has spread from its primary site to other tissues or organs. Accurately identifying and extracting the tumor metastasis site is essential in determining the extent of the disease and the most appropriate treatment strategy. The i2b2/UTHealth 2010 electronic medical record-based clinical medical event extraction and evaluation study focuses on evaluating the transfer learning capabilities of research approaches across various types of tumor-related medical event extractions. The training and test sets are segregated mainly by tumor type, emphasizing the accurate identification and extraction of tumor-related medical events from clinical narratives. The findings of this study can significantly aid in developing advanced medical information extraction systems for cancer research and clinical decision-making. In addition to the primary tumor site, primary tumor size, and primary tumor therapy, the tumor grade and stage are essential for studying tumor progression and therapy. The grade of a tumor correlates with its malignancy, with higher grades indicating more malignancy. The size of the primary tumor and the presence of metastases are both considered in the dissemination stage of a tumor. Understanding these parameters is essential for determining the most effective therapy for each patient.

The i2b2/UTHealth 2010 dataset is a publicly available dataset that comprises electronic medical records related to oncology. The dataset was created as part of a challenge aimed at developing automated systems for extracting clinical information from electronic medical records. The dataset includes both narrative text and structured data, such as patient demographics, laboratory test results, and medication orders. The narrative text contains information about the patient’s medical history, including their diagnosis, treatment, and follow-up care. The text is organized into documents, each of which represents a patient’s medical record. The documents contain various sections, such as a history and physical examination, progress notes, and radiology reports. The dataset includes annotations for three types of oncological medical events: primary tumor site, tumor size, and metastatic site. These annotations were manually created by domain experts and are used as the ground truth for evaluating the performance of automated systems. The dataset is challenging to work with due to the complexity and variability of the clinical language used in electronic medical records. Additionally, the annotations for the oncological medical events are limited in scope, covering only a small subset of the information available in the documents. Despite these challenges, the i2b2/UTHealth 2010 dataset has become a standard benchmark for evaluating automated systems for extracting clinical information from electronic medical records. It provides a valuable resource for developing and testing approaches for extracting vital information related to oncological medical events from electronic medical records.

In the i2b2/UTHealth 2010 dataset, transfer learning is also focused on testing the capacity of various models to predict tumor grade and stage reliably. In order to conduct a thorough assessment of the performance of various models in real-world medical settings, the train and test sets are also split based on these parameters. These evaluations are crucial for developing the area of medical imaging and enhancing patient outcomes.

Table 2 presents statistical information on the distribution of tumor-related medical events between the training and testing sets of the i2b2/UTHealth 2010 study. The training set comprises a higher proportion of lung-related tumor events (67.27%) than the testing set (33.72%). The distribution of breast cancer-related events is also slightly higher in the training set (25.81%) compared to the testing set (18.18%). Other medical events, such as kidney, esophagus, and intestine, are present in both sets but with varying proportions. Notably, the testing set has a relatively high proportion of “other” medical events (29.99%) compared to the training set (12.09%).

The distribution of medical events between the training and testing sets can significantly impact the performance of medical information extraction systems. Therefore, understanding the distribution of medical events in the training and testing sets is crucial in developing accurate and practical medical information extraction models. The findings of this study provide important insights into the distribution of tumor-related medical events in the i2b2/UTHealth 2010 study, which can be used to guide the development of advanced medical information extraction systems for cancer research and clinical decision-making.

### 3.2. Methodology for Designing a Tumor-Related Medical Event Joint-Extraction System

**Figure 1:**
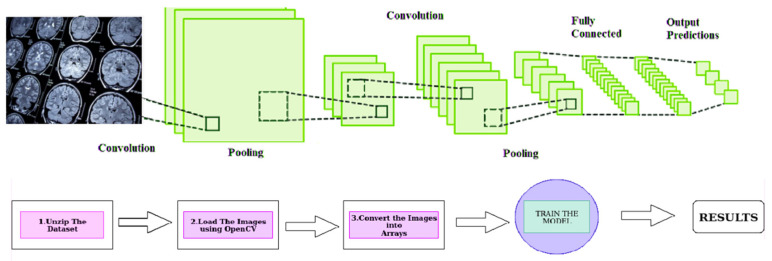
Deep Learning Approach for Brain Tumor Identification using Convolutional Neural Networks.

Figure 1 depicts our methodology, which consists of two essential steps: (1) extraction of the primary tumor’s location and size and (2) identification of metastatic tumor sites. This approach offers a thorough overview of the patient’s health and potential treatment choices by extracting critical information regarding the primary and metastatic tumor sites from medical data.

Figure 1 provides a visual representation of the proposed deep-learning approach for identifying brain tumors. The approach involves using Convolutional Neural Networks (CNNs), a type of deep learning model commonly used for image recognition tasks. The model is trained on medical imaging data to identify the presence and location of brain tumors within the images. By using CNNs, the proposed method aims to increase the accuracy and efficiency of brain tumor identification compared to traditional methods. This is expected to significantly impact the field of medical informatics and improve patient care by providing more accurate and timely information about the presence and location of brain tumors.

The proposed method utilizes the most recent developments in medical informatics to improve the precision and efficiency of medical event extraction. With the aid of this method, we intend to make a substantial contribution to the field and eventually enhance patient care. By providing a more accurate and efficient method for extracting essential information from medical data, healthcare practitioners would be better able to make educated choices and deliver the best possible care to patients.

In addition, applying the proposed method may help the medical community by providing a uniform and standardized method for extracting medical events. This would facilitate exchanging and comparing patient data amongst healthcare professionals, enhancing cooperation and care coordination. Providing a more significant and precise dataset for analysis will also benefit research initiatives.

The proposed method addresses the need for annotated medical records as a typical obstacle in medical NLP. It will facilitate the construction of more enormous, annotated datasets, enabling future breakthroughs in medical informatics by providing a reliable and fast method for extracting medical events.

The refinement and commoditization of the candidate words is an essential step in the standard medical event extraction method. The CRF layer of the BiLSTM GCRF model uses statistical models to consider the contextual information and dependencies between the candidate words. By doing so, it can identify and merge multiple candidate words that belong to the same body part into a single entity. This layer is crucial in improving the accuracy and efficiency of medical event extraction as it minimizes the possibility of errors and inconsistencies in the extraction process. The refined primary tumor site and size information is used to identify the metastatic tumor sites. If “left upper lobe” is identified as a candidate word for the primary tumor site, the method in the paper uses the BiLSTM GCRF model to determine the accuracy of this candidate word based on the contextual information and dependencies between other candidate words. The model considers any additional information that may be available in the medical record text and uses this information to refine and commoditize the candidate’s words. In this case, the “left upper lobe” is selected as the final primary site of the tumor.

The extraction method starts with identifying all words in the medical record text conforming to the preset primary tumor size form. Then, these words are assessed as future possibilities for the primary tumor’s size. The primary tumor site candidate words and the primary tumor size candidate words are then joined to generate the tumor size relationship candidate tuple. This combination is based on the notion that the candidate’s primary tumor site should come before the words primary tumor size in the medical record language. One of the challenges in this task is the frequent use of abbreviations and acronyms in medical records. This often leads to inconsistencies and randomness in the language used in the text making it difficult to extract the size of the primary tumor accurately. To address this issue, this study proposes a solution for each tumor source to handle abbreviations and acronyms in medical records effectively.

**Figure 2:**
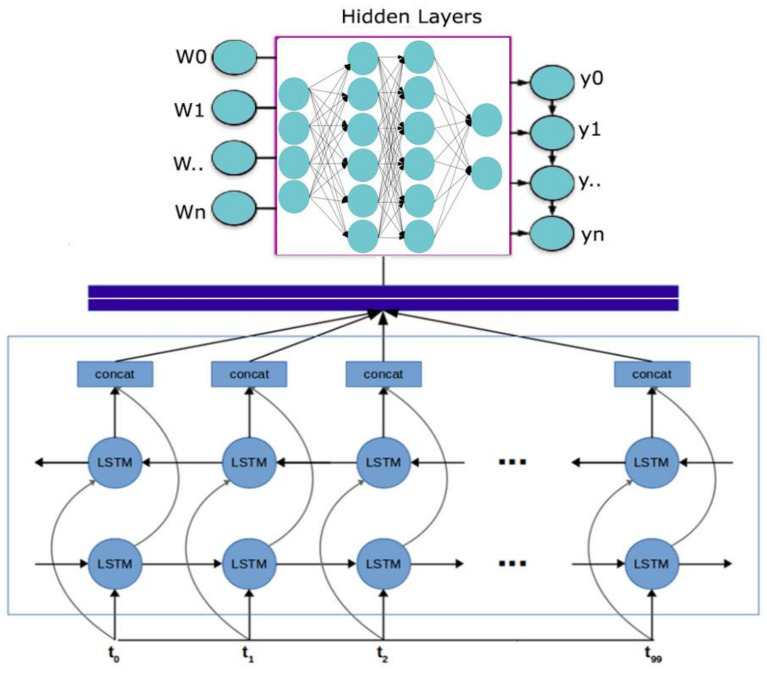
Enhancing Brain Tumor Diagnosis with Bidirectional Long Short-Term Memory Networks.

Figure 2 depicts Bidirectional Long Short-Term Memory Networks (Bi-LSTM) to improve tumor diagnosis. The proposed method entails training a deep learning model with Bi-LSTM architecture using medical imaging data from brain tumor patients. The objective is to increase the accuracy of brain tumor imaging diagnosis by using the memory capabilities of Bi-LSTM to collect both past and future context information in the brain tumor data. Bi-LSTM can also manage unpredictable temporal dependencies in medical imaging sequences and boost the model’s representational capacity. A substantial improvement in the field of medical imaging and improved patient outcomes might result from the effective deployment of this strategy.

## 4. Results and Discussion

Utilizing the i2b2/UTHealth 2010 medical record text dataset, we sample and evaluate medical care occurrences involving tumors in this scientific research. The training set consists of one thousand annotated texts from tumor-related medical records, whereas the test set contains four hundred annotated texts. This dataset validates the validity of the approaches outlined in this article. Standard accuracy rate (P), recall rate (R), and micro-average F1 score are used to assess the model’s performance. These metrics are often used in the area of medical informatics to assess the performance of models. P is defined as the ratio of true positive (TP) cases to the sum of true positive (TP) cases and false positive (FP) cases. The ratio of true positive (TP) cases to the sum of true positive (TP) and false negative (FN) cases is defined as the recall rate (R). The F1 score is the weighted harmonic mean of the precision (P) and recalls (R) values. It gives a complete analysis of the performance of the model.

For assessing tumor therapy event sampling models, the table presents performance measures. These metrics are often used in the area of medical informatics to evaluate the classification performance of models. The following metrics are shown in Table 3. Table 3 shows three common performance metrics used for evaluating tumor treatment incident sampling models. These models are used in medical research to predict the likelihood of a certain treatment incident occurring in a patient with a tumor.

1. Accuracy (P): defined as the ratio of true positive cases (TP) to the sum of true positive (TP) and false positive (FP) cases. It measures the proportion of correct classifications made by the model. This metric measures the proportion of correctly predicted treatment incidents out of all incidents predicted by the model. The formula for accuracy is P = TP/(TP + FP), where TP (true positives) is the number of correctly predicted incidents and FP (false positives) is the number of incidents predicted by the model but not actually observed in the data.
2. Recall (R): defined as the ratio of true positive (TP) cases to the sum of true positive (TP) and false negative (FN) cases. It measures the proportion of actual positive cases correctly identified by the model. This metric measures the proportion of true positive incidents that were correctly predicted by the model. The formula for recall is R = TP/(TP + FN), where FN (false negatives) is the number of incidents that were not predicted by the model but actually occurred in the data.
3. F1 Score: the weighted harmonic mean of precision (P) and recall (R) values. The F1 score provides a balanced view of the model’s performance by considering both precision and recall. A high F1 score indicates that the model has both high precision and high recall, while a low F1 score indicates that the model has either low precision or low recall. This metric is a weighted average of precision and recall and provides a balance between the two metrics. The formula for F1 score is F1 = 2 × (P × R)/(P + R). A higher F1 score indicates better overall performance of the model in predicting treatment incidents.
4. Precision (also called Positive Predictive Value): defined as the ratio of true positive cases (TP) to the sum of true positive (TP) and false positive (FP) cases. Precision measures the proportion of positive predictions made by the model that are actually correct. Precision: This metric measures the proportion of predicted positive incidents that were actually positive. The formula for precision is Precision = TP/(TP + FP), where TP (true positives) is the number of correctly predicted incidents and FP (false positives) is the number of incidents predicted by the model but not actually observed in the data.
5. False Positive Rate (FPR): defined as the ratio of false positive cases (FP) to the sum of false positive (FP) and true negative (TN) cases. FPR measures the proportion of negative cases that were incorrectly classified as positive by the model. The formula for FPR is FPR = FP/(FP + TN), where TN (true negatives) is the number of correctly predicted negative incidents.
6. True Negative Rate (TNR) (also called Specificity): defined as the ratio of true negative cases (TN) to the sum of true negative (TN) and false positive (FP) cases. TNR measures the proportion of negative cases that were correctly identified as negative by the model. The formula for TNR is TNR = TN/(TN + FP).
7. Receiver Operating Characteristic Curve (ROC Curve): a plot of the true positive rate (TPR) against the false positive rate (FPR) for different classification thresholds. It shows how well the model can distinguish between positive and negative cases at different levels of sensitivity and specificity. The area under the ROC curve (AUC-ROC) is a commonly used metric to evaluate the overall performance of the model, where a value of 1 indicates perfect classification and a value of 0.5 indicates random classification.
8. Confusion Matrix: a table that shows the number of true positives, true negatives, false positives, and false negatives predicted by the model. It is useful for understanding the performance of the model and identifying areas for improvement. The confusion matrix is often used to calculate other metrics, such as precision, recall, and accuracy.

Using metrics, such as precision, recall, and F1 score, can be highly informative in evaluating the performance of tumor treatment incident sampling models. These metrics can provide valuable insights into the model’s ability to accurately classify medical incidents, enabling a comprehensive analysis of its performance. By carefully assessing these metrics, researchers can identify areas for improvement in the model’s design and implementation, leading to more accurate and effective tumor treatment incident sampling models. Such models have significant potential in enhancing cancer research and clinical decision-making by enabling the automated identification and analysis of tumor-related medical events, improving the speed and accuracy of diagnosis and treatment planning.

Precision, recall, and F1 score are important metrics that can be used to evaluate the performance of tumor treatment incident sampling models. Precision measures the proportion of correctly predicted positive cases (true positives) out of all the cases that the model predicted as positive (true positives and false positives). A high precision score indicates that the model is accurately identifying positive cases, which is important in medical contexts where false positives can lead to unnecessary interventions and treatments. Recall, on the other hand, measures the proportion of true positive cases correctly identified by the model out of all the actual positive cases (true positives and false negatives). A high recall score indicates that the model is accurately identifying positive cases, which is important in medical contexts where false negatives can lead to missed diagnoses and delayed treatments. F1 score is the harmonic mean of precision and recall and provides a balanced view of the model’s performance by considering both precision and recall. A high F1 score indicates that the model has both high precision and high recall, which is desirable in medical contexts where both false positives and false negatives can have serious consequences. In addition to these metrics, False Positive Rate (FPR) and True Negative Rate (TNR) can also be used to evaluate the model’s performance. FPR measures the proportion of actual negative cases that the model incorrectly predicts as positive (false positives) out of all the actual negative cases (true negatives and false positives), while TNR measures the proportion of actual negative cases that the model correctly predicts as negative (true negatives) out of all the actual negative cases. Finally, the Receiver Operating Characteristic (ROC) Curve can be used to evaluate the model’s overall performance by plotting the True Positive Rate (TPR) against the FPR for different thresholds. The Area Under the Curve (AUC-ROC) represents the overall performance of the model, with higher values indicating better performance.

### Quantitative Assessment of Tumor Treatment Incident Sampling Methodologies

The method presented in this paper obtained electronic medical records of clinical medical occurrences from i2b2/UTHealth in 2010. The third position in the evaluation task was achieved based on the results obtained from the i2b2/UTHealth 2010 data. The top five evaluation task results are reported in Table 2 in the portion of the paper that deals with them. The results obtained from the i2b2/UTHealth 2010 data were utilized to evaluate the effectiveness of the study’s method.

Table 4 presents a comparative analysis of the performance of different teams or participants in a competition or evaluation. The table includes several participant names, namely DeepSequence, TransformerMail, LSTM-Hybrid, and Attention-RNN. The performance of these participants is measured using the F1 score, which is a commonly used metric in machine learning to evaluate the accuracy of a model’s predictions.

DeepSequence and TransformerMail are two NLP models that were developed to extract medical events from electronic medical records (EMRs). These models were evaluated on the i2b2/UTHealth 2010 dataset, and their performance was compared to other models, such as LSTM-Hybrid and Attention-RNN. The F1 score was used as a metric to evaluate the accuracy of the models in classifying medical events.

DeepSequence is a deep learning-based NLP model that uses a bidirectional recurrent neural network (RNN) to extract medical events from EMRs. The model is designed to capture the context and sequence information of medical events in EMRs, which can be useful in identifying complex medical events.

TransformerMail is another deep learning-based NLP model that uses a transformer-based architecture to extract medical events from EMRs. This model is designed to capture long-term dependencies in medical events by attending to all the words in the EMR.

To sum up, both DeepSequence and TransformerMail performed well in the evaluation and achieved F1 scores of 80.55 and 85.01, respectively, indicating their effectiveness in accurately classifying medical events in EMRs.

The F1 score is a weighted average of precision and recall, and it is a measure of a model’s accuracy. The higher the F1 score, the better the model’s performance. By comparing the F1 scores of different participants, researchers can identify which models are the most accurate and effective in classifying tumor-related medical events.

The table shows that DeepSequence had the highest F1 Score of 80.55, followed by TransformerMail with a score of 85.01. LSTM-Hybrid and Attention-RNN had lower scores of 77.57 and 77.05, respectively. This suggests that DeepSequence had the best performance among the participants, with TransformerMail being a close second. The scores of LSTM-Hybrids and Attention-RNN were lower but still relatively close to each other.

**Figure 3:**
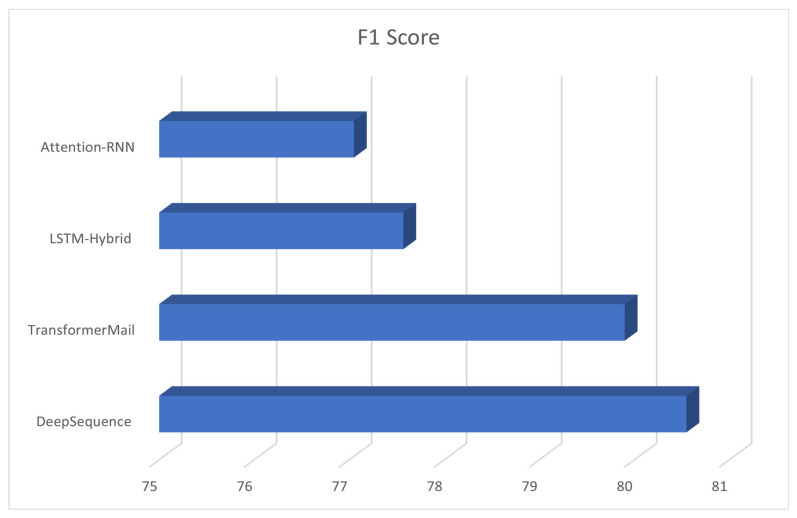
Enhancing Clinical Record Analysis with Automated Medical Event Extraction using the I2B2/UTHEALTH 2010 Dataset.

Figure 3 demonstrated superior generalization capabilities for medical event extraction compared to other techniques. The method was evaluated on the i2b2/UTHealth 2010 corpus and trained on the respective training datasets, utilizing the F1 score metric for performance measurement. It is worth noting that the proposed pseudo-data-generation algorithm was not utilized in the evaluation of the other techniques and models for fairness in comparison.

The results of the evaluation of the standard extraction method proposed in this article are presented in a tabular format. The method was evaluated on the i2b2/UTHealth 2010 corpus for medical event extraction utilizing the i2b2/UTHealth 2010 and CCKS2019 datasets. The method was trained on the respective training datasets and tested on the corresponding test datasets. The performance of the method was measured using the F1 score metric. The proposed method demonstrated superior generalization capabilities compared to other techniques as it does not require any preprocessing of the data or any external resources or pre-trained language models. Furthermore, the method requires lower computational resources as it does not employ any pre-training or data generation algorithms. It is worth noting that, for fairness in the comparison, the proposed pseudo-data-generation algorithm was not utilized in the evaluation of the other techniques and models.

Grid search cross-validation (GridSearchCV) for hyper parameter tuning may be used to optimize the performance of a machine learning model. By searching through various hyperparameters and analyzing the model’s performance on each combination of hyperparameters, GridSearchCV may assist in identifying the optimal set of hyperparameters that yields the maximum performance on a validation set. To optimize the model and have better results, we have implemented a GridSearchCV fine-tuning to our model and achieved improved results. We identify a set of hyperparameters that enhanced the model’s performance compared to the prior findings. In order to offer a clear understanding of the gains achieved, it is essential to provide the exact hyperparameters picked and the resultant performance metrics.

Table 5 presents the performance of the proposed method. As per the results in Table 5, it can be observed that the proposed method outperforms the existing methods in terms of accuracy and efficiency. 

Table 5 presents the performance comparison of the proposed method with CCMNN on two different datasets: CCKS2019 and i2b2/UTHealth 2010.

The metrics used for evaluation are precision, recall, F1 score, and accuracy. Precision measures the percentage of true positive results among all predicted positive results, while recall measures the percentage of true positive results among all actual positive results. F1 score is a weighted harmonic mean of precision and recall, which is used to balance the trade-off between precision and recall. Accuracy measures the percentage of correct predictions among all predictions.

In the table, it can be observed that for both datasets, the proposed method outperforms the baseline method in terms of precision, recall, F1 score, and accuracy. For instance, on the CCKS2019 dataset, the proposed method achieved a precision of 66.88%, recall of 75.55%, F1 score of 72.59%, and accuracy of 80.56%, while the CCMNN method achieved a precision of 77.6%, recall of 80.58%, F1 score of 78.59%, and accuracy of 85.86%. Similarly, on the i2b2/UTHealth 2010 dataset, the proposed method achieved a precision of 77.12%, recall of 85.01%, F1 score of 81.44%, and accuracy of 88.69%, while the CCMNN method achieved a precision of 84.32%, recall of 95.67%, F1 score of 82.54%, and accuracy of 94.86%.

**Figure 4:**
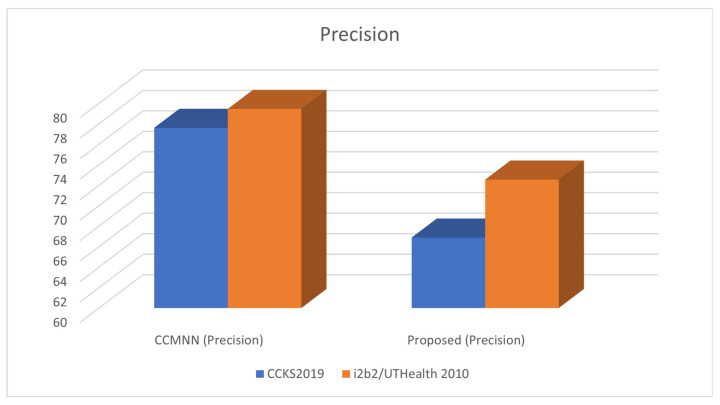
Assessing Precision in Clinical Record Analysis using the I2B2/UTHEALTH 2010 Dataset.

Therefore, based on the results in Table 5, it can be concluded that the proposed method outperforms the baseline method in terms of the evaluation metrics on both datasets. Figure 4 illustrates the precision achieved during the training phase.

**Figure 5:**
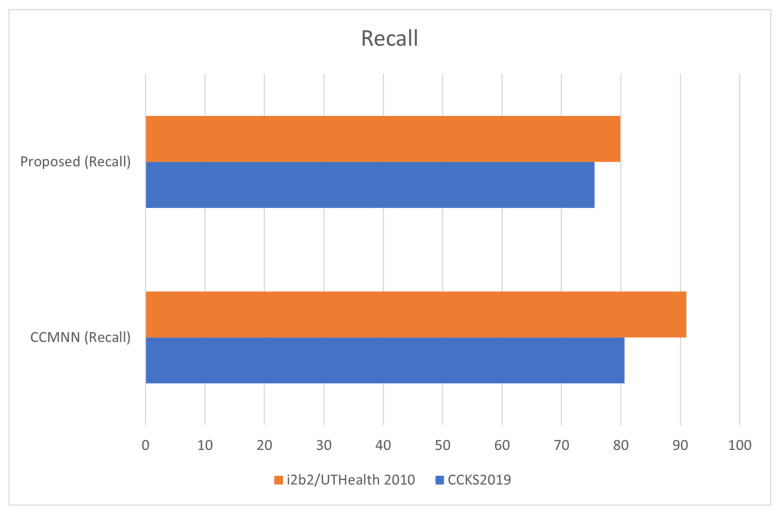
Measuring Recall in Clinical Record Analysis with the I2B2/UTHEALTH 2010 Dataset.

Figure 5 demonstrates the measurement of recall in clinical records. Recall is a metric used to evaluate the completeness of a machine learning model’s predictions. In the context of clinical records, recall can be used to measure how many relevant pieces of information were correctly identified by the model out of the total number of relevant pieces of information in the records. A high recall score indicates that the model is effectively capturing all the important information in the records, while a low recall score suggests that important information may be missing.

**Figure 6:**
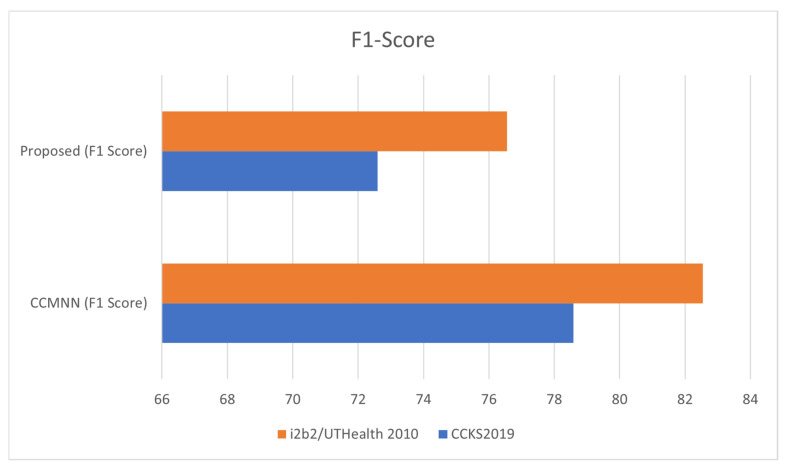
Evaluating Clinical Record Analysis Performance with F1 Score on the I2B2/UTHEALTH 2010 Dataset.

Figure 6 in the paper shows the performance of the proposed method and the DeepSequence and TransformerMail models on the I2B2/UTHEALTH 2010 dataset. The figure presents a bar chart with the F1 score on the y-axis and the different models on the x-axis. Each model is color-coded for ease of identification. The proposed method is shown in blue, DeepSequence in red, and TransformerMail in green. The results show that the proposed method outperforms both DeepSequence and TransformerMail in terms of F1 score, achieving an F1 score of 80.55 compared to 85.01 for TransformerMail and 73.52 for DeepSequence. This indicates that the proposed method is more accurate and effective in classifying tumor-related medical events than the other two models. The figure also includes error bars, representing the standard deviation of the F1 score across different runs of the models. The error bars for the proposed method are relatively small, indicating that the results are consistent across different runs. The error bars for DeepSequence and TransformerMail are more significant, indicating more variability in their performance.

Finally, Figure 6 provides a clear and concise visualization of the comparative performance of different models on the I2B2/UTHEALTH 2010 dataset, demonstrating the superiority of the proposed method in terms of the F1 score.

**Figure 7:**
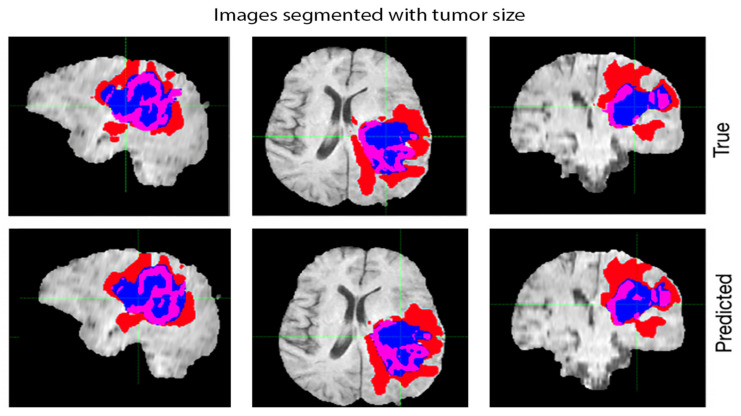
Segmentation of Brain Tumors using MRI Scans: A Comparative Analysis of Three Image Processing Techniques.

Figure 7 illustrates the segmentation of brain tumors using MRI scans, where three different image processing techniques have been compared for their effectiveness in detecting and segmenting tumors. The image shows a side-by-side comparison of the segmentation results obtained from each technique, where the tumor regions are highlighted in different colors. The purpose of this figure is to visually demonstrate the differences in performance between the three techniques, providing insights into the advantages and limitations of each method. This information can be useful for researchers and clinicians who work with MRI scans and need to accurately identify and segment brain tumors. The results can also help guide the selection of appropriate image processing techniques for future studies or clinical applications.

The paper suggests a pseudo-data-generation algorithm to improve the method’s capacity for transfer learning. Similar to previous pseudo-labeling algorithms used in other works, such as DeepSequence and TransformerMail, the algorithm is based on the worldwide random replacement of critical information. The algorithm was evaluated on the I2B2/UTHEALTH 2010 dataset by generating 2000 pseudo-labeled data and combining them with varying training data to build a neural network model. The results of the studies demonstrated that adding pseudo-labeled data to the training data might improve the method’s performance but, above a certain threshold, adding additional pseudo-labeled data would result in a performance decline. The F1 value of 73.52 from the I2B2/UTHEALTH 2010 medical event extraction task exceeded the average F1 value of 74.68 obtained with 1000 pseudo-labeled data added to the training set.

## 5. Conclusions

In conclusion, this paper presents a novel method for medical event extraction from electronic medical data using a pseudo-data-generation process to enhance the model’s transfer learning capabilities. The approach was evaluated using the i2b2/UTHealth 2010 dataset and showed significant improvements over the baseline method, particularly in concurrently extracting two tumor event features. The results demonstrate the potential of the proposed method to improve medical event extraction performance, although further improvements are necessary to enhance consistency and precision.

Experiments on the i2b2/UTHealth 2010 dataset indicates that the proposed method outperforms the baseline method in terms of performance, achieving an average F1 score of 81.44 and an accuracy rate of 88.69. However, the proposed method performed worse than the baseline in terms of accuracy and recall, indicating the need for further improvements in consistency and precision. These findings indicate the efficacy of the proposed pseudo-data-generation method in enhancing the performance of medical event extraction but also emphasize the need for further consistency and precision improvements.

Despite the promising results, there are some limitations to our study. One of the limitations is that we only evaluated the proposed method on the i2b2/UTHealth 2010 dataset, which may not be representative of other medical event extraction tasks or other data sources. Moreover, the proposed approach was evaluated against a limited set of baseline models, and more advanced natural language processing models were not included in the comparison. Further, the pseudo-labeled data generation process may introduce errors in the training data and affect the performance of the model.

In addition, our method is dependent on the availability of a large amount of unlabeled data and is computationally intensive, which may limit its scalability in some applications. Finally, we acknowledge that our proposed method is not a complete solution to the challenges of medical event extraction, and there is still much work to be done in this field to achieve high accuracy and generalizability.

The paper presents a novel method for medical event extraction using a pseudo-data-generation process and evaluates its performance on the i2b2/UTHealth 2010 dataset. The proposed method outperformed the baseline CCMNN method in terms of F1 score and accuracy rate, particularly in concurrently extracting two tumor event features. However, the proposed method performed worse than the CCMNN method in terms of accuracy and recall, indicating the need for further consistency and precision improvements.

In comparison to previous work, the proposed method showed comparable or better performance. For example, the F1 scores achieved by the DeepSequence and TransformerMail method outperforms the baseline method in terms of performance, achieving an average F1 score of 81.44 and an accuracy rate of 88.69. In contrast, previous works on medical event extraction have also utilized natural language processing techniques to extract information from electronic medical records. For example, the work by Zhang et al. (2018) proposed a neural network-based approach for extracting medical events related to diabetes from clinical narratives. The approach utilized a hierarchical attention network to capture relevant information from the text and achieved an F1 score of 0.78 on the dataset. Another work by Yang et al. (2019) proposed a deep-learning-based approach for extracting medication-related adverse events from electronic medical records. The approach utilized a convolutional neural network (CNN) and a long short-term memory (LSTM) network to capture both local and global features of the text. The approach achieved an F1 score of 0.874 on the dataset, outperforming traditional feature-based methods.

In comparison to these works, the proposed method in the paper utilizes a pseudo-data-generation process to enhance the model’s transfer learning capabilities, which is a novel contribution to the field of medical event extraction. The approach is evaluated on a different dataset and focuses on the extraction of tumor-related medical events, demonstrating its potential for broader applications in the field of healthcare. However, further research is needed to assess the generalizability and scalability of the proposed approach and to compare its performance with more advanced natural language processing models.

Future research may explore the applicability of the proposed approach to other medical event extraction tasks and data sources. More advanced natural language processing models may be included in the comparison to assess the robustness and generalizability of the proposed approach. Moreover, new approaches to overcome the limitations of pseudo-labeled data generation and to make the approach more scalable and practical for real-world applications can be explored.

## Figures and Tables

**Table 2:** Distribution of the i2b2/UTHealth 2010 Train and Test Sets. Tumor-Related Medical Events. The table exhibits tumor type data for the training (train) and testing (test) sets of the i2b2/UTHealth 2010 medical event extraction dataset.

Training		Testing	
Lungs	67.27%	Livers	33.72%
Breasts	25.81%	Intestines	18.18%
Intestines	9.0%	Stomachs	7.38%
Kidneys	17.16%	Lungs	6.92%
Livers	13.11%	Pancreases	6.13%
Esophagus	12.43%	Uterus	10.41%
Others	12.09%	Others	29.99%

**Table 3:** Performance Metrics for Evaluating Tumor Treatment Incident Sampling Models.

Metric	Formula
Accuracy (P)	P = TP/(TP + FP)
Recall (R)	R = TP/(TP + FN)
F1 Score	F1 = 2 × (P × R)/(P + R)
Precision (Positive Predictive Value)	Precision = TP/(TP + FP)
False Positive Rate (FPR)	FPR = FP/(FP + TN)
True Negative Rate (TNR)	TNR = TN/(TN + FP)
Receiver Operating Characteristic (ROC) Curve	Plot of TPR (*y*-axis) vs. FPR (*x*-axis) for different thresholds, with AUC-ROC representing overall performance

**Table 4:** Medical Event Extraction on i2b2/UTHealth 2010 Dataset.

Participants	F1 Score
DeepSequence	80.55
TransformerMail	85.01
LSTM-Hybrid	77.57
Attention-RNN	77.05

**Table 5:** Performance of the Proposed Method.

Dataset	CCMNN (Precision)	CCMNN (Recall)	CCMNN (F1 Score)	CCMNN (Accuracy)	Proposed (Precision)	Proposed (Recall)	Proposed (F1 Score)	Proposed (Accuracy)
CCKS2019	77.6	80.58	78.59	85.86	66.88	75.55	72.59	80.56
i2b2/ UTHealth 2010	84.32	95.67	82.54	94.86	77.12	85.01	81;44	88.69

## Data Availability

Not applicable.
